# VHL-recruiting PROTAC attenuates renal fibrosis and preserves renal function via simultaneous degradation of Smad3 and stabilization of HIF-2α

**DOI:** 10.1186/s13578-022-00936-x

**Published:** 2022-12-19

**Authors:** Jiayi Yang, Yuyi Ruan, Dan Wang, Jinjin Fan, Ning Luo, Huiting Chen, Xiaoyan Li, Wei Chen, Xin Wang

**Affiliations:** 1 Department of Nephrology, The First Affiliated Hospital, Sun Yat-Sen University, Guangzhou, 510080 China; 2 NHC Key Laboratory of Clinical Nephrology (Sun Yat-Sen University) and Guangdong Provincial Key Laboratory of Nephrology, Guangzhou, 510080 China

**Keywords:** PROTACs, Smad3, HIF-2α, Renal fibrosis

## Abstract

**Background:**

Renal fibrosis is the pathological foundation of various chronic kidney diseases progressing to end stage renal failure. However, there are currently no nephroprotective drugs targeted to the fibrotic process in clinical practice. Proteolytic targeting chimeras (PROTACs), which reversibly degrade target proteins through the ubiquitin–proteasome pathway, is a novel therapeutic modality. Smad3 is a key pathogenic factor in fibrogenesis while HIF-2α exhibits prominent renal protective effects, which is the natural substrate of von Hippel–Lindau (VHL) E3 Ligase. We hypothesied the construction of VHL-recruiting, Smad3-targeting PROTAC might combine the effects of Smad3 degradation and HIF-2α stabilization, which not only improving the clinical efficacy of PROTAC but also avoiding its potential off-target effects, could greatly improve the possibility of its translation into clinical drugs.

**Methods:**

By joining the Smad3-binding small molecule compound (SMC) to VHL-binding SMC with a linker, we designed and synthesized a Smad3-targeting, VHL-based PROTAC. The effects of this PROTAC on targeted proteins were verified both in vitro and in vivo. The toxicity and pharmacokinetic (PK) evaluations were conducted with both male and female mice. The renal protection effects and mechanism of PROTAC were evaluated in unilateral ureteral obstruction (UUO) and 5/6 subtotal nephrectomy (5/6Nx) mouse model.

**Results:**

By optimizing the linker and the Smad3-binding SMC, we got a stable and high efficient PROTAC which simultaneously degraded Smad3 and stabilized HIF-2α both in vivo and in vitro*.* The acute toxicity evaluation showed a pretty large therapeutic window of the PROTAC. The prominent renal protection effects and its underlying mechanism including anti-fibrosis and anti-inflammatory, improving renal anemia and promoting kidney repair, had all been verified in UUO and 5/6Nx mouse model.

**Conclusion:**

By accurate combination of PROTAC targeted protein and E3 ligase, we got a Smad3-targeting, VHL-recruting PROTAC which caused Smad3 degradation and HIF-2α stabilization effects simultaneously, and led to the strong renal function protection effects.

**Supplementary Information:**

The online version contains supplementary material available at 10.1186/s13578-022-00936-x.

## Background

Proteolysis Targeting Chimeras (PROTACs) is a novel therapeutic modality, which targets protein to degradation via ubiquitin–proteasome pathway (UPP). A typical PROTAC comprises a moiety specifically binding to a protein of interest (POI), a recognition moiety for E3 ubiquitin ligase and a chemical linker connecting these two parts together [1]. PROTACs mediate the degradation of POIs through proteasome by hijacking the activity of E3 ubiquitin ligases to ubiquitinate the POI [2]. Thus, the high efficacy of PROTAC may not only induce the degradation of POI, but also hinder the ubiquitination and degradation of E3 ligase’s natural substrate (ELNS), which could be either beneficial or harmful.

**Fig. 1:**
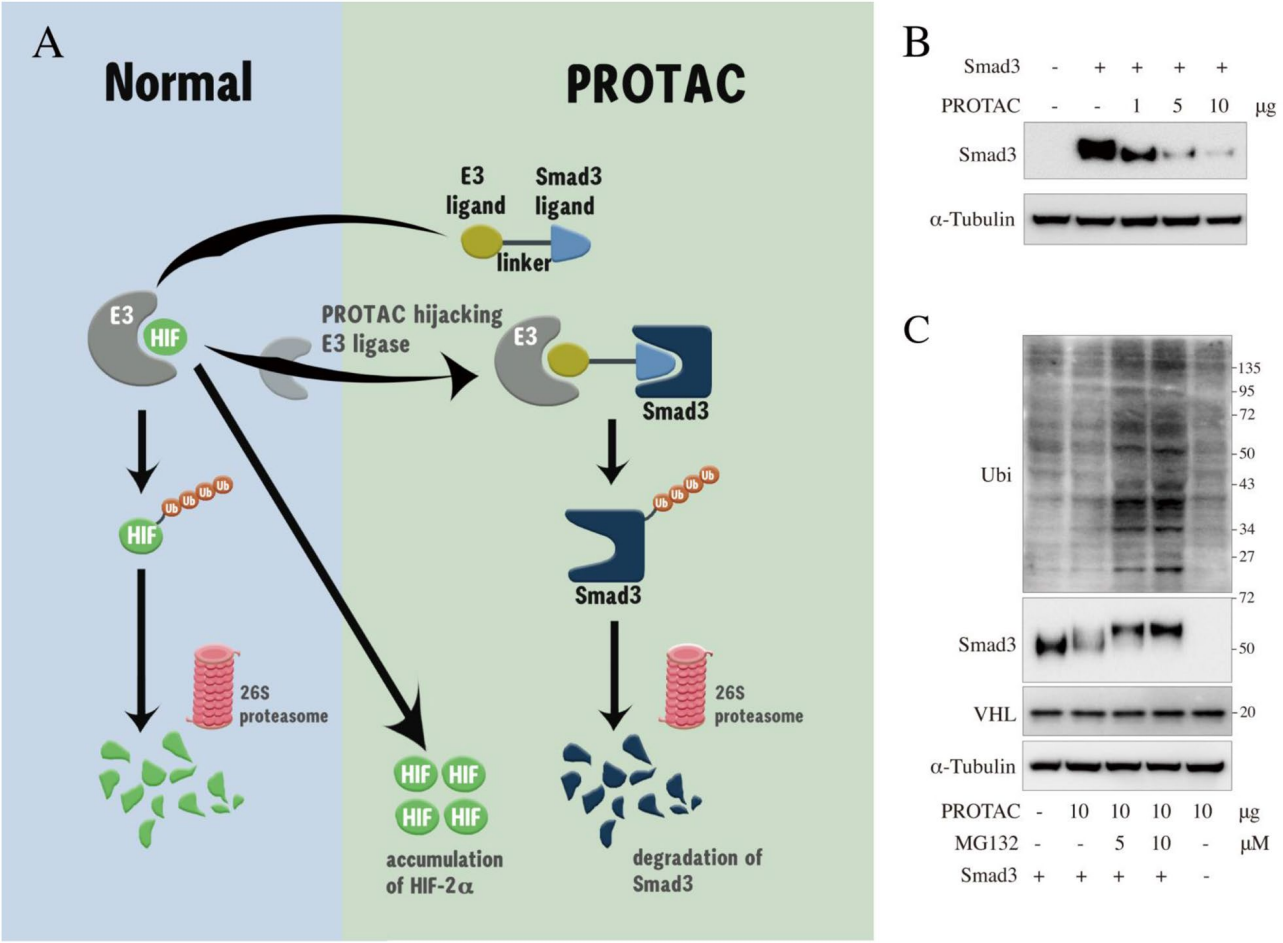
Mechanism of VHL-recruiting, Smad3-targeting PROTAC. **A** Schematic of PROTAC-mediated Smad3 degradation and HIF-2α stabilization. **B** The impact of different amount of PROTAC on the degradation of Smad3 recombinat protein was investigated with ACHN whole cell lysate expressing high level of VHL. **C** PROTAC-mediated polyubiquitination and degradation of Smad3 protein via ubiquitin–proteasome pathway. The degradation and ubiquitination on Smad3 protein induced by PROTAC was compared in the absence and presence of MG132 (a specific proteasome inhibitor) with ACHN whole cell lysate expressing high level of VHL

Smad3 has long been widely recognized as the core signal protein of fibrosis. We previously combined computer virtual screening with surface plasmon resonance (SPR) technology and screened out a small molecule compound (SMC) that specifically binds to Smad3. Based on this SMC we constructed a PROTAC which could degrade Smad3 through UPP [3]. Knowing that HIF-2α plays a strong reno-protective role in the progression of chronic kidney disease, and protein von Hippel–Lindau (VHL) is a HIF specific E3 ligase [4], we hypothesized that a new PROTAC, connected this Smad3-binding SMC and a VHL-binding SMC with a linker, might have two effects at one time: the degradation of Smad3 as well as the stabilization of HIF-2α (Fig. 1A). To verify our hypothesis, we constructed and optimized this VHL-recruting, Smad3-targeting PROTAC, then verified its multiple reno-protective functions both in vitro and in vivo.

## Results

### PROTAC degrades Smad3 via ubiquitin–proteasome pathway

The lysates of human renal carcinoma (ACHN) cells were used to detect the degradation of recombinant Smad3 by PROTAC in vitro since ACHN cells contained very high pVHL protein level [3].Western blot results showed that the degradation of Smad3 was related to the amount of PROTAC (Fig. 1B). Then, MG132, a well-known proteasome inhibitor, were added to the ACHN cell lysates in different concentrations and the degradation of Smad3 was rescued accordingly. A slight increase in the molecular weight of Smad3 was observed, which might suggest the ubiquitination of Smad3 (ubiqutin protein molecular weight is 8 KD). To further verify this hypothesis, ubiquitin antibody was used and as we expected that significantly increased ubiquitination bands were only visible in the lanes where MG132 were added along with the PROTAC (Fig. 1C).

### Chemical synthesis and optimization of PROTAC

Generally, inhibitors of POI are used as binding moiety of PROTAC. But this strategy is not appropriate for Smad3. Smad3 can only be ubiquitinated after its phosphorylation, while its inhibitor works by hindering it’s phosphorylation. Thus based on our previous work, a new identified Smad3 binding SMC was used as POI binding moiety [3]. Another SMC which binded to VHL specifically was chosen as E3 recognition ligand [5].

**Fig. 2:**
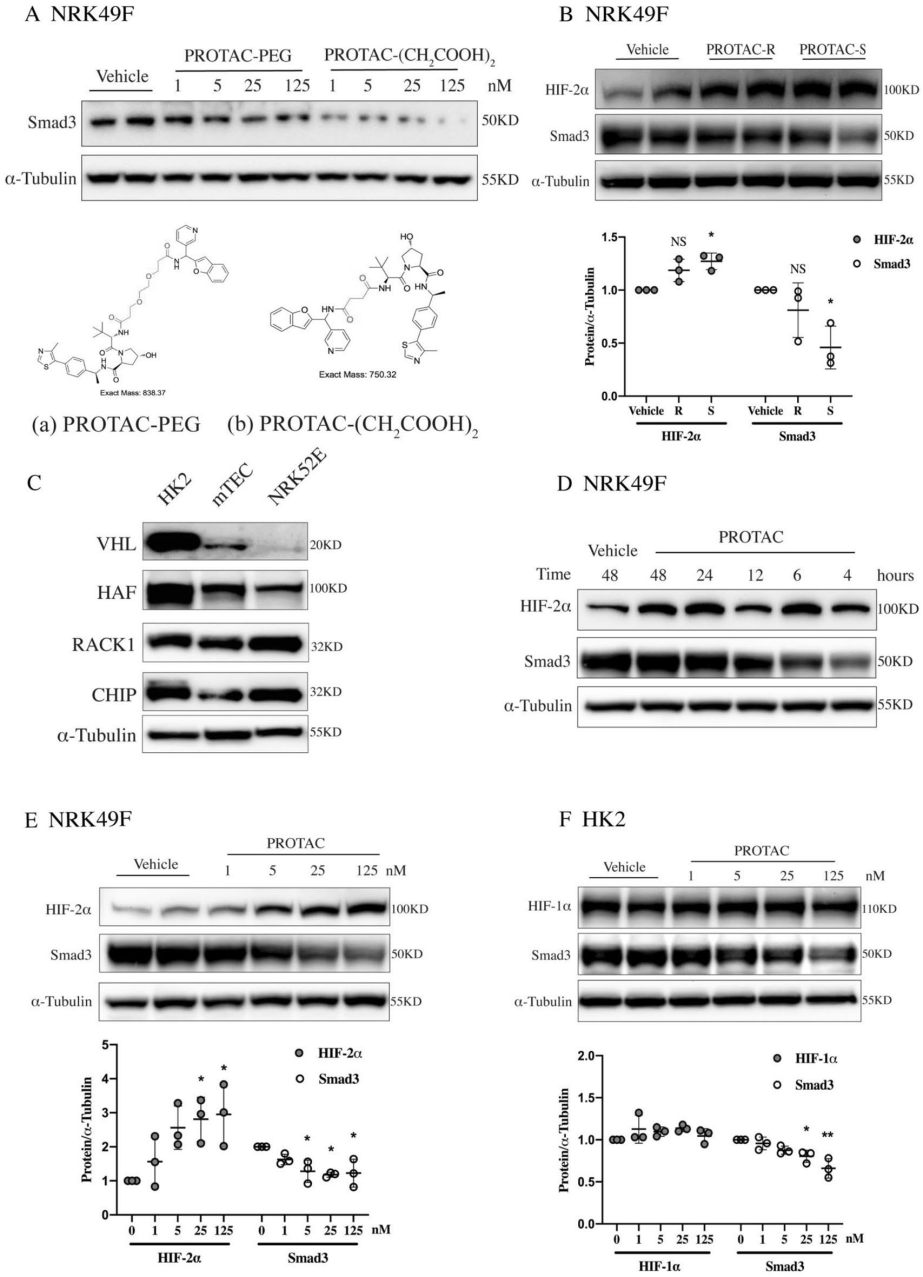
PROTAC degrades Smad3 and stabilizes HIF-2α but not HIF-1α in vitro. **A** Treatment of NRK-49F cells with PROTAC of two linkers: **a** PROTAC-PEG and **b** PROTAC-(CH_2_COOH)_2_. **B** Treatment of NRK-49F cells with 25 nM PROTAC of two charities: PROTAC-R and PROTAC-S and the plot represented three independent experiments (n = 3). **C** The expression levels of different E3 ligases in human, mouse and rat renal tubular cells (HK2, mTEC, NRK52E). **D** Time course treatment of NRK-49F cells with 25 nM of PROTAC. **E** Treatment of NRK-49F cells with PROTAC of different concentration and the plot represented three independent experiments (n = 3). **F** Treatment of HK2 cells with PROTAC of different concentration and the plot represented three independent experiments (n = 3). PEG = Polyethylene glycol; (CH_2_COOH)_2_ = Succinic acid. Data are means ± SD; NS = not significant, *P < 0.05, **P < 0.01

Considering that different linkers may also affect the efficiency of the PROTAC [6], PROTACs were synthesized with two different linkers: (a) PROTAC-Polyethylene Glycol (PEG): longer linker with better flexibility and (b) PROTAC-(CH_2_COOH)_2_: shorter linker may have better efficiency in ubiquitination (Fig. 2A). After identification of connection sites between small molecules and linker (Additional file 1: Fig. S1), chemical synthesis of PROTAC was completed by Ontores (Zhejiang, China). The purity and the structure of chemicals were confirmed with the High Performance Liquid Chromatography (HPLC) and Mass Spectrum (MS), respectively (Additional file 1: Fig. S2A, B). NRK-49F cells were stimulated with PROTAC-PEG or PROTAC-(CH_2_COOH)_2_ in various concentrations. The degradation of Smad3 was more prominent in PROTAC-(CH_2_COOH)_2_ stimulated group with a much lower effective concentration (Fig. 2A). Thus, (CH_2_COOH)_2_ was chosen as the liker.

Since the Smad3 binding SMC is a chiral molecule with R and S configurations, then PROTAC-R and PROTAC-S were all synthesized and tested in NRK-49F cells. The results showed that PROTAC-S was more potent than PROTAC-R not only on the degradation of Smad3 but also on the stabilization of HIF-2α (Fig. 2B). Thus, PROTAC-(CH_2_COOH)_2_ in S chirality was chosen for the following in-vitro and in-vivo study and referred as PROTAC. The PROTAC is a white lyophilized powder, with molecular weight of 750.8, which was confirmed in blinded-side again with MS (Additional file 1: Fig. S2C).

### PROTAC degrades Smad3 and stabilizes HIF-2α but not HIF-1α in vitro

Since the expression of HIF-1α and HIF-2α in kidney are cell-type specific (HIF-1α has only been found in tubular cells and podocytes, whereas HIF-2α could be found in fibroblasts and endothelial cells) [7], both rat kidney interstitial fibroblasts (NRK-49F) and tubule cells were chosen for in vitro experiment. The high expression level of E3 ligase VHL in NRK-49F cells was confirmed in our previous study [3]. Then the protein level of VHL was tested in human, mouse and rat renal tubule cell lines (HK2, mTEC, NRK-52E) respectively. HK2 cells (human renal tubule cell lines) showed the highest protein level of VHL and thus was chosen for the following study (Fig. 2C).

In NRK-49F cells, significant but transient down regulation of Smad3 protein level and more lasting upregulation of HIF-2α protein level were found after the stimulation of PROTAC (Fig. 2D). PROTAC treatment significantly reduced Smad3 protein levels and elevated HIF-2α protein levels dose-dependently with the effective concentration as low as 25 nM (~ 2 ng/mL, Fig. 2E).

In HK2 cells, Smad3 protein showed significantly dose-dependent reduction without any HIF-1α protein accumulation after PROTAC treatment (Fig. 2F). To clarify the reason, other reported E3 ligases targeting HIF-1α, including hypoxia associated factor (HAF), receptor of activated protein kinase C (RACK1) and carboxyl terminus of Hsc70-interacting protein (CHIP), were all tested in kidney tubular cells. The high expression levels of those non-VHL HIF-1α E3 ligases suggested that there were non-VHL dependent degradations of HIF-1α, which might interpret the absent accumulation effect of PROTAC on HIF-1α (Fig. 2C, F).

### Safety assessment of PROTAC in mice

**Fig. 3:**
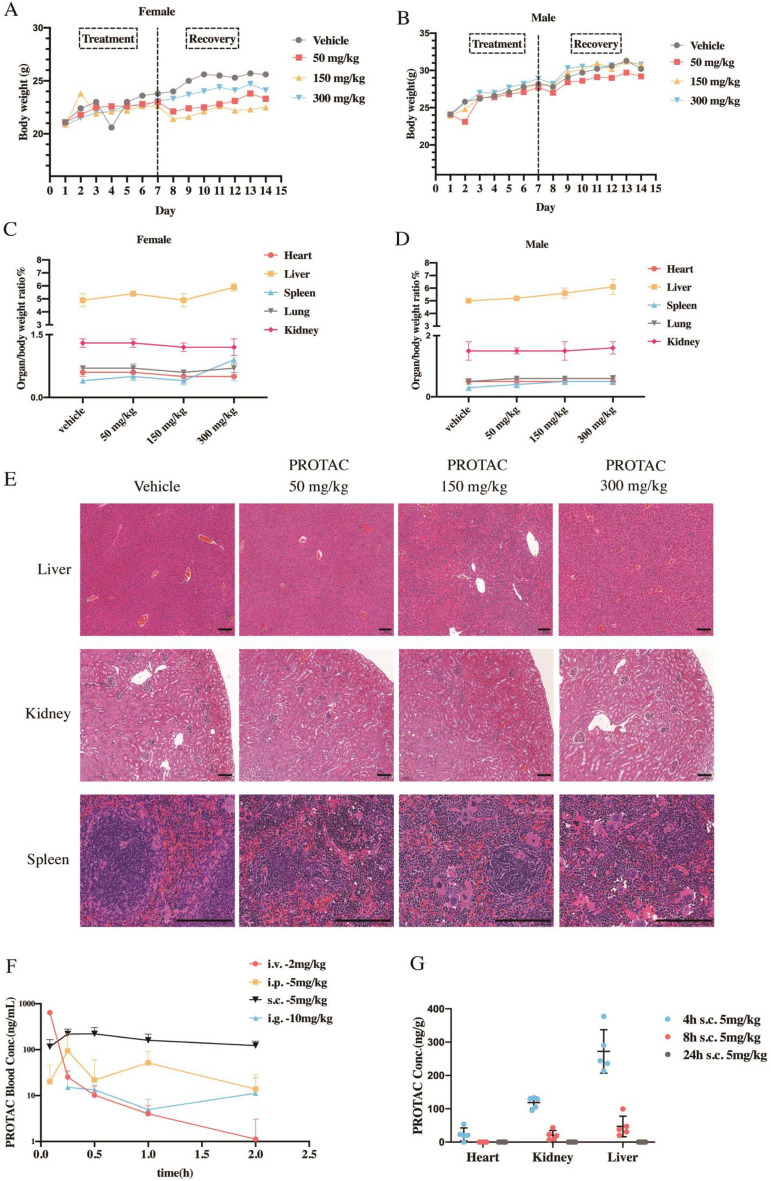
Toxicity and pharmacokinetic assay of PROTAC in mice. The effects on body weight (**A**, **B**) and organ/body weight ratio (**C**, **D**) of female and male mice s.c. administered with vehicle or 50, 150 and 300 mg/kg of PROTAC. **E** Representative HE staining of different organs of female and male mice administered with vehicle or 50, 150, and 300 mg/kg of PROTAC. Scale bars: 100 μm. **F** PK profile of PROTAC in mice after a single dose with the indicated routes and concentrations (n = 3/per group). **G** The concentration of PROTAC in relevant tissue compartments after subcutaneous administration (5 mg/kg) at indicated times (n = 5/per group). Data are means ± SD. Conc. = concentration, i.v. = intravenous, i.p. = intraperitoneal, s.c. = subcutaneous, i.g. = gavage

Subacute and chronic toxicity experiments in mice showed no signs or behavioral abnormalities during the treatment or recovery period. All mice grew normally with the gradually increased body weights (Fig. 3A, B). The hematological and biochemical data showed no difference among groups (Additional file 2: Tables S1, S2). The organ/body weight ratio of heart, liver, spleen, lung and kidney did not show any significant changes between PROTAC treatment and normal control groups (Fig. 3C, D). The liver, kidney and spleen tissues were stained with hematoxylin–eosin (HE). In PROTAC-treated mice, only increased nucleated cells in the spleen were observed, but no splenomegaly or changes in red blood cell (RBC) counts (Fig. 3C-E, Additional file 2: Table S1). The toxicity evaluation in vivo showed that PROTAC had a pretty large therapeutic window.

### Pharmacokinetic attributes of PROTAC in mice

**Fig. 4:**
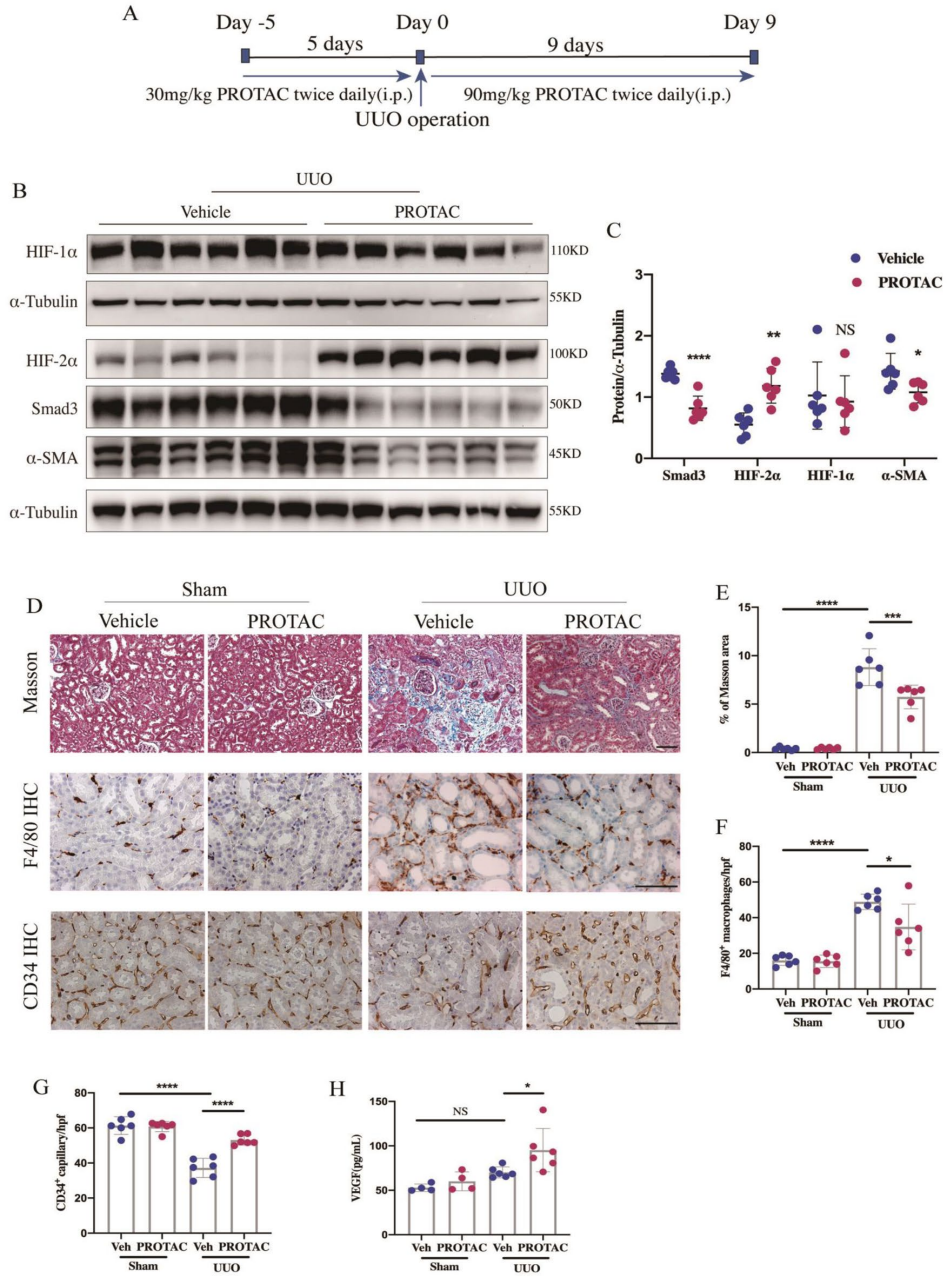
PROTAC attenuates renal fibrosis and inflammation and enhances peritubular capillaries density of UUO mice. **A** Experimental design of evaluating the therapeutic effect of PROTAC on UUO animal models. **B** Western blot analyses of HIF-1α, HIF-2α, Smad3 and $\alpha$-SMA expression and **C** quantification of them in UUO mice (n = 6/per group). **D** Representative Masson’s trichrome staining, F4/80 and CD34 immunochemical staining of renal sections among indicated groups. **E** The positive-stained area of Masson’s trichrome was quantitatively measured ($\times$400 magnification, n = 6/per group). **F** The count of F4/80^+^ macrophages/HPF of indicated groups (n = 6/per group). **G** The count of CD34^+^ capillary/HPF of indicated groups (n = 6/per group). The serum level of **H** VEGF was determined in indicated groups (n = 4–6/per group). Veh = vehicle, i.p. = intraperitoneal, UUO = unilateral ureteral obstruction, IHC = immunohistochemistry, HPF = high power field (magnification: $\times$400). Scale bar = 500 μm. Data are means ± SD, NS = not significant, *P < 0.05, **P < 0.01, ***P < 0.001, ****P < 0.0001

The pharmacokinetic (PK) data showed that PROTAC administered by i.p. 5 mg/kg resulted in plasma levels of PROTAC significantly above the predicted efficacious concentration [C_max_ = 94.1 ng/mL, efficacious concentration = 25 nM (~ 2 ng/mL)], but the clearance of PROTAC was fast with the concentrations falling over the next 3 h to ~ 1.6 ng/mL (T_1/2_ = 0.481 h, Fig. 3F, Additional file 2: Table S3). Thus, in consideration of the aggressive and intense fibrotic progress as well as the key profibrotic effects of Smad3 in UUO model, intraperitoneal PROTAC administration with a high dose and high frequency was used (Fig. 4A).

**Fig. 5:**
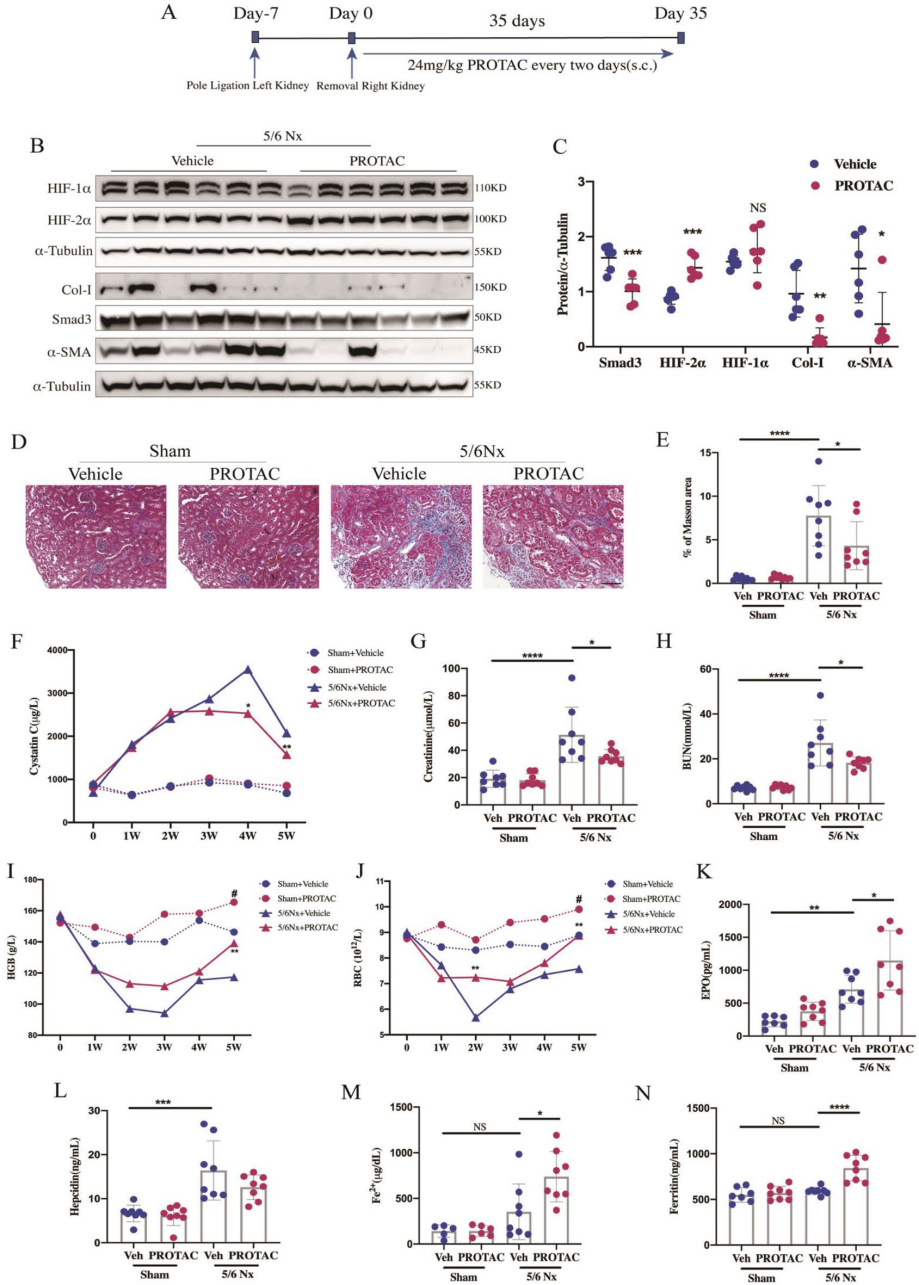
PROTAC attenuates renal fibrosis and ameliorates renal function and renal anemia of 5/6Nx mice. **A** Experimental design of evaluating the therapeutic effect of PROTAC on 5/6Nx animal models. **B** Western blot analyses of HIF-1α, HIF-2α, Smad3, collagen-I and α-SMA expression and **C** quantification of them in 5/6Nx mice (n = 6/per group). **D** Representative Masson’s trichrome staining of renal sections among indicated groups. Scale bar: 500 μm. **E** The positive-stained area of Masson’s trichrome was quantitatively measured ($\times$400 magnification, n = 8/per group). **F** Trajectory of Cystatin C of indicated groups at indicated times (n $\ge$ 6/per group) and renal function was evaluated with **G** serum creatinine and **H** serum BUN after 5 weeks of vehicle or PROTAC treatment (n = 8/per group). Trajectory of **I** HGB and **J** RBC counts of indicated groups at indicated times (n $\ge$ 6/per group, **P < 0.01 compared between PROTAC-treated and vehicle-treated mice in 5/6Nx group. ^#^*P* < 0.05 compared between PROTAC-treated and vehicle-treated mice in Sham group). **K** The serum level of EPO was determined in indicated groups. The situation of iron mobilization was indicated with serum level of **L** Hepcidin, (M) Fe^2+^ and **N** Ferritin of indicated groups after 5 weeks of treatment. Veh = vehicle, 5/6Nx = 5/6 nephrectomy model, HGB = hemoglobin, RBC = red blood cells. Data are means ± SD. NS = not significant, *P < 0.05, **P < 0.01, ***P < 0.001, ****P < 0.0001

A single subcutaneous administration of 5 mg/kg PROTAC resulted in a 221 ng/mL peak concentration and more lasting time above effective concentration (C_max_ = 221 ng/mL, T_1/2_ = 1.58 h, Fig. 3F, Additional file 2: Table S3). In consideration that both Smad3 and HIF-2α play prominent effects in the process of CKD, and other HIF-stabilizers are given mostly every 2 days for a long period [8–10], PROTAC administrated subcutaneously with intermittent dosing (24 mg/kg, every 2 days for 5 weeks) was chosen for 5/6Nx mouse model (Fig. 5A).

The exposure level of PROTAC in the relevant tissue compartments after subcutaneous administration (5 mg/kg) was also explored. Peak concentrations in heart, kidney and liver were achieved at 4 h after administration and then eliminated over time (Fig. 3G).

### PROTAC degrades Smad3 and stabilizes HIF-2α but not HIF-1α in vivo

The protein and mRNA levels of HIF-1α and Smad3 increased significantly in obstructed kidneys of UUO mice compared with the sham controls (Additional file 1: Figs. S3, S4). Treatment of PROTAC could significantly down-regulate Smad3 protein level (~ 40%) but had no obvious effects on the protein level of HIF-1α in both obstructed kidneys of UUO mice and remnant kidneys of 5/6Nx mice. PROTAC administration could significantly increase the expression of HIF-2α level to approximately 2 times compared to the vehicle control in both models (Figs. 4B, C, 5B, C). Moreover, real-time quantitative PCR indicated that the chage of Smad3 and HIF-2α in protein level by PROTAC was not caused from the transcription level change (Additional file 1: Fig. S4).

### PROTAC attenuates renal fibrosis and inflammation in both UUO and 5/6Nx mice

Masson’s trichrome staining showed a severe renal fibrosis after UUO and 5/6Nx surgery, while PROTAC treatment significantly ameliorated the fibrosis in both models (Figs. 4D, E 5D, E). Concomitant with the fibrosis, the increased macrophages infiltration in tubulointerstitium, determined by immunohistochemistry staining of F4/80^+^ cells, was observed in UUO mice model, which were significantly mitigated with the PROTAC treatment (Fig. 4D, F). The extra cellular matrix (ECM) deposition, assessed by collagen I, as well as the epithelial–mesenchymal transition (EMT) of tubular epithelial cells, assessed by myofibroblast marker α-SMA, were all significantly reduced after PROTAC treatment in UUO and 5/6Nx mice model (Figs. 4B, C, 5B, C).

### PROTAC restores peritubular capillary density in UUO mice

Peritubular capillaries (PTCs) were observed by immunohistochemistry staining of CD34, a typical marker of endothelial cells. As presented in Fig. 4D, PTCs could be easily recognized in the interstitial area between tubules. UUO surgery led to the loss of PTCs while PROTAC administration could restore it (Fig. 4G). As the most important regulator of angiogenesis, one of the main targeting genes of HIF-2α, the level of vascular endothelial growth factor (VEGF) in serum was tested with ELISA. The significant elevation of serum VEGF in UUO model group with the treatment of PROTAC should explain the restoration of PTCs (Fig. 4H).

### PROTAC preserves renal function in 5/6Nx mice

To explore the effect of PROTAC on renal function, 5/6Nx mice model was established. One week after the surgery, significant reduction of renal function was observed in 5/6Nx group and the renal function getting worse gradually after that, evidenced by the increasing serum cystatin C, creatinine and blood urea nitrogen (BUN). Compared to the vehicle-treated 5/6Nx mice, PROTAC-treated 5/6Nx mice showed a similar damaged renal function in the first 2 weeks after intervention, but a much better renal function after that, evidenced by the much lower levels of serum cystatin C, creatinine and BUN (Fig. 5F-H).

### PROTAC improves renal anemia and iron utilization in 5/6Nx mice

One week after the surgery, the 5/6Nx mice presented a reduction in RBC count and Hemoglobin (HGB) concentration and became significant on week 2, indicating the success of the renal anemia model. PROTAC intervention caused improvement in anemia on week 2 and got full correction on week 5. In the Sham group, PROTAC intervention could also significantly increase the levels of RBC count and HGB of the mice without anemia (Fig. 5I, J).

Erythropoietin hormone (EPO), the main regulator of erythropoiesis, is one of the most important targeted genes of HIF-2α. Compared with the Sham group, serum EPO level was markedly increased in 5/6Nx group, which was consistent with the partial recovery of anemia. Compared with those 5/6Nx mice treated with vehicle, 5/6Nx mice with PROTAC treatment showed a more significant elevation in serum EPO levels (Fig. 5K). Hepcidin, a strong negative regulator of iron metabolism, was considered as the main reason of iron utilization disorder in renal anemia. ELISA results showed that there were no difference in serum levels of Fe^2+^ and ferritin between Sham and 5/6Nx groups, but a significantly elevated level of hepcidin in 5/6Nx group could be observed. However, with the PROTAC treatment, hepcidin had a tendency of attenuation, accompanied with a significant increase of Fe^2+^ and ferritin (Fig. 5L-N). The transcriptional levels of duodenal cytochrome b (Dcytb), divalent metal transporter1 (DMT1) and ferroportin1 (FPN1), all classic downstream target genes of HIF-2α related to intestinal iron absorption ability [11], were also detected. A significant reduction of Dcytb, DMT1 and FPN1 could been seen in 5/6Nx mice indicating the impairment of intestinal iron absorption capacity. PROTAC treatment could significantly elevate the mRNA levels of Dcytb, DMT1 and FPN1 (Additional file 1: Fig. S5) which may work with the elevated EPO to improve the anemia.

## Discussion

With the application of SMCs as the moiety of PROTAC, the performance of PROTAC improved and more E3 would be hijacked to POI, which may cause the accumulation of ELNS. To verify this hypothesis, we constructed a PROTAC with two SMCs targeting Smad3 (POI) and VHL (E3) separately. Then a high efficient PROTAC was obtained by optimizing it with different linkers and different chiral configurations of Smad3 connecting SMC. Smad3 degradation and HIF-2α stabilization effects of this PROTAC were verified both in vitro and in vivo. Its strong anti-fibrosis activity was confirmed in mouse UUO model, a classic renal interstitial fibrosis model, and prominent renal function protection as well as renal anemia treatment effects were verified in 5/6Nx mouse model, a widely used chronic renal failure model.

Unlike the traditional inhibitors, PROTAC targets protein to degradation via UPP and works in an “event-driven” mode, which means a transient interaction with the target protein is sufficient for PROTAC to trigger protein degradation. As a result, one PROTAC may repeatedly bind to numerous POIs and thus a much lower concentration is needed for a lasting effect [12]. PROTAC has been reported to degrade various target proteins, even some traditional “untargeted proteins” [13]. During the past few years, with the widely application of SMCs targeting E3 as ligand to construct PROTAC, the effectiveness of PROTAC in degrading target proteins has been greatly improved. Knowing that all the degradation happened within a limited space and a limited time, exhaustion of specific E3 targeted by PROTAC might happen, then the accumulation of ELNS would not be avoided. As the main regulator of hypoxia, transcription factor HIF-α regulates its downstream hypoxia related proteins expression within a few minutes after the sensation of hypoxia. The basis of this extremely rapid response is that HIF-α protein levels are regulated by an oxygen-dependent ubiquitination process [14]. Once this high efficient PROTAC hijacks VHL for the degradation of Smad3, it will inevitably lead to the hindrance of HIF-α ubiquitination and then accumulation. In fact, the feature of PROTAC leading accumulation of ELNS was also reported with other E3 ligase, which exert a synergistic effects combied with the degradation of POI [15]. So we supposed purposeful choosing moiety to target specific E3, the effect of ELNS stabilization might magnify the therapeutic benefits by working together with the degradation of POIs. Refer to this strategy, we designed and constructed a novel Smad3-targeting, VHL-recruting PROTAC and confirmed its effects on anti-renal fibrosis and renal function-improving.

Finding a satisfactory treatment for renal fibrosis has been a long-standing challenge [16]. TGF-β1/Smad3 signaling is a common pathway involving in renal fibrosis, which contributes to extracellular matrix (ECM) deposition [17], tubular epithelial–mesenchymal transition (EMT) [18] and kidney inflammation [19]. Therapeutic strategies targeting Smad3, including genetically inactivation and traditional small molecule inhibitors, have all been confirmed their extraordinary anti-fibrosis and renal protection effects [20–23]. However, Smad3 as an indispensable factor in the regulation of a broad spectrum of biological processes, its complete blockage may lead to serious side effects [24, 25]. Traditional inhibitors work by preventing Smad3 from localizing to target locus, thus high systemic drug concentration and continuous exposure are often required in order to get sufficient functional inhibition, which may cause side effects and off-target toxicity during therapy [26]. In contrast, PROTAC could directly degrade Smad3 protein without interference in its mRNA level and could transcriptionally downregulate its fibrotic response, leading to a temporary and reversible inhibition mechanism over traditional inhibitors (Additional file 1: Figs. S4, S6).

Importantly, the function of PROTAC is targeting E3 level dependent. Kidney-targeting specificity of our PROTAC is attributed to VHL, an E3 ligase, which mainly found in brain and kidney [27]. Besides, the function of our PROTAC is also Smad3-level dependent, which means that higher level of Smad3 leads to higher efficacy, and thus PROTAC has not much effects on normal tissue in consideration of the limited expression level of Smad3 under normal condition. This is consistent with our result showing that PROTAC treatment had no significant effects on the target protein levels in the control kidneys of UUO mice (Additional file 1: Fig. S3). Therefore, the therapeutic effects of PROTAC was more profound in injured kidneys, because of the higher Smad3 level. These results provide the possibility that patients with kidney diseases might get more benefits from PROTAC treatment.

Notably, the expression of HIF-α subtypes in kidney is cell-type specific [28]. Thus, after identifying the expression level of VHL, we needed to confirm the effects of PROTAC on the expression level of HIF-1α and HIF-2α using tubular cells (HK2) and fibroblasts (NRK-49F) separately (Fig. 2E, F). In addition to cell-type specificity, HIF-1α and HIF-2α also show differences in their function. HIF-1α is indicated as a pro-fibrotic factor since it can increase collagen expression [29, 30]. In contrast, HIF-2α shows protective effects against renal fibrosis [31–35]. Moreover, the interdependence between HIF-1α and TGF-β1/Smad3 signal pathway has been well studied [36]. The pro-fibrotic function of HIF-1α is Smad3-dependent. HIF-1α-mediated induction of collagen expression relies on the expression of Smad3, in which both Smad3 and HIF-1α are needed to translocate in the nucleus to participate in the formation of transcription-regulatory complexes to elicit HIF-1α dependent fibrogenesis [37, 38]. Whereas, there was no any evidence of a similar ineraction between HIF-2α and Smad3 signaling pathway. One study had investigated the potential relationship between Smad3 and HIF-2α by overexpressing HIF-2α, but there were no detectable effects had been found on the p-Smad3 levels [39]. Based on these characteristics, the degradation of Smad3 could alleviate fibrosis not only by inhibiting TGF-β1/Smad3 signaling, but also blocking the pro-fibrotic effect of HIF-1α, and thus only the benefits of HIF-2α left with the treatment of PROTAC. This accurate combination of target proteins could maximize the therapeutic value of the PROTAC.

**Fig. 6:**
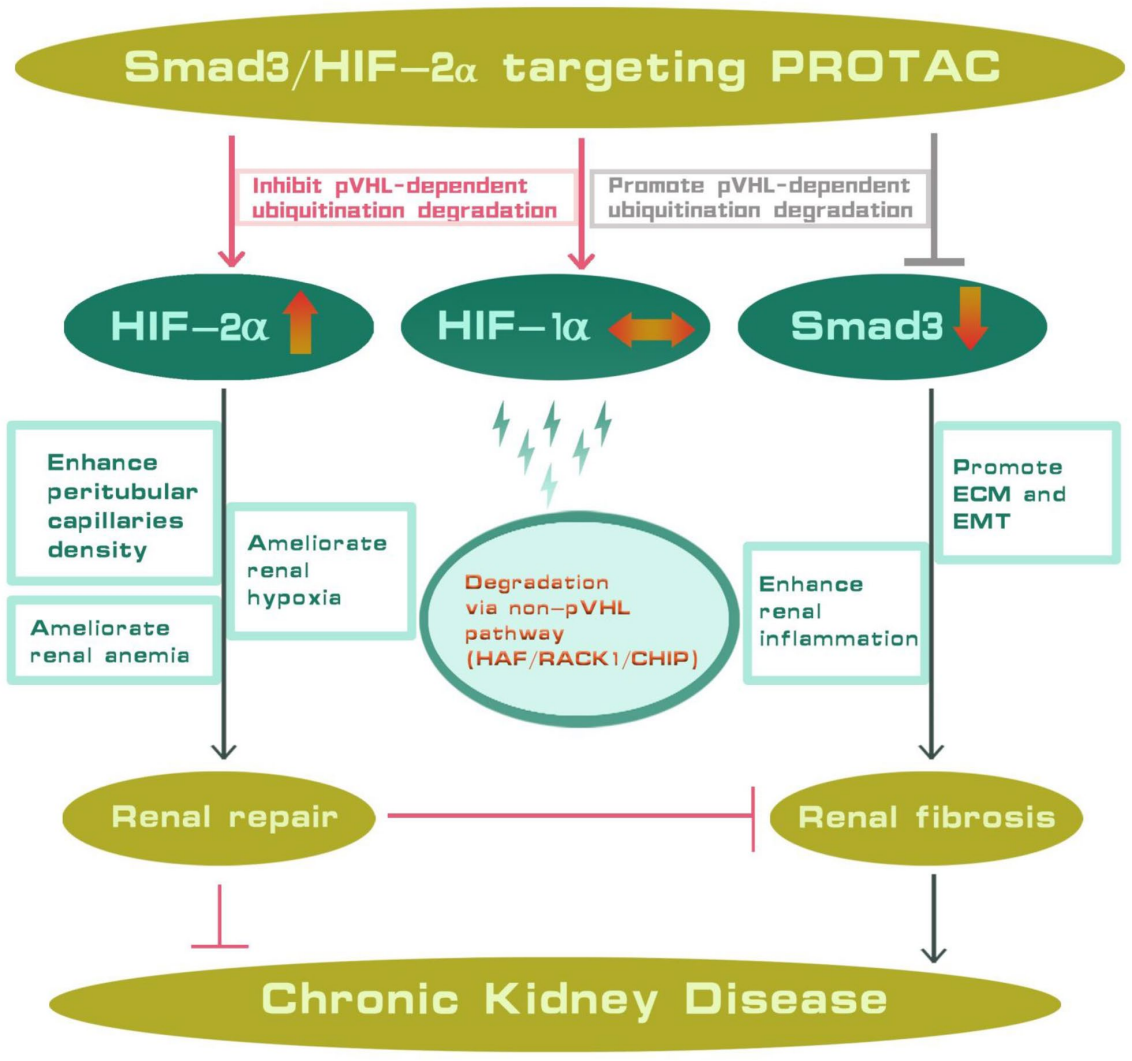
VHL-based Smad3-targeting PROTAC protects from chronic kidney disease through simultaneously reducing renal fibrosis and promoting renal repair. ECM = excessive extracellular matrix, EMT = epithelial–mesenchymal transition

Moreover, other than VHL E3 ligase, the degradation of HIF-1α can also rely on other ubiquitin ligases, such as RACK1, HAF and CHIP [40–43], which are highly expressed in renal tubular cells (Fig. 2C). On the contrary, the degradation of HIF-2α is restricted to VHL pathway. Once VHL is occupied by PROTAC, the continuously produced HIF-2α has no way to degrade, leading to its prominent accumulation. While the degradation of HIF-1α, depending on other ubiquitin ligases, continues (Fig. 6). A similar phenomenon has also been observed in HIF-PHD 1–3 pan inhibitors which mainly up-regulate the level of HIF-2α downstream proteins, but not HIF-1α [44, 45]. Therefore, treatment with PROTAC could ameliorate fibrogenesis due to the degradation of Smad3 together with the stabilization of HIF-2α. PROTAC treatment leads a composite protective effects in kidney fibrosis.

HIF-2α also works as a key regulator in adult erythropoiesis, by activating the *EPO* gene [46] and increasing iron utilization, both had been demonstrated in HIF-PHD inhibitors previously [10, 47]. Similar effects were also found in our study (Fig. 5K-N). PROTAC treatment could also increase the transcriptional level of Dcytb, DMT1 and FPN1 in duodenum and elevate EPO expression in kidney, while had no obvious effects on the expression level of HIF-1α target genes (Transferrin and Transferrin receptor 1) in liver (Additional file 1: Fig. S5). Thus, with elevated EPO expression and obvious iron mobilization, renal anemia in 5/6Nx mice was partially corrected after only 2 weeks of PROTAC treatment and got full correction on week 5 (Fig. 5I, J), which was more quickly than HIF-PHD inhibitors did [48]. Moreover, extramedullary erythropoiesis was observed in the spleen when PROTAC was used in high dosage in our toxicity assay (Fig. 3E). Erythropoiesis mainly takes place in the bone marrow, while the spleen may also produce RBCs under certain conditions. The PHD2-deficient mice displayed HIF-2α-induced erythrocytosis, accompanied by excessive RBCs, thrombocytopenia and extramedullary erythropoiesis in the spleen, which effects were all HIF-1α-independent [49]. Thus, the extramedullary erythropoiesis we observed might be induced by increased EPO via HIF-2α activation. The relatively short administration time of our toxicity assay might account for the absence of change of peripheral RBCs (Additional file 2: Table S1).

Although this PROTAC has shown great success in renal fibrosis mice models, its exactly working mechanism has not been fully elucidated. Since Smad3 is ubiquitously expressed and our study has been mainly focused on renal interstitium, the evaluation of Smad3 and HIF-2α protein levels with PROTAC treatment in other cells/tissues were still needed in the future study. Besides, as a precursor of medicine, whether it has long-term toxic effects still needs to be explored.

## Conclusions

Our study showed a VHL-recruiting PROTAC comprising a Smad3-binding SMC and a VHL-binding SMC connected with a (CH_2_COOH)_2_ linker. This Smad3-targeting VHL-based PROTAC has Smad3 degradation and HIF-2α stabilization effects simultaneously, leading to the strong renal function protection effects (Fig. 6). Besides, this study not only provides a promising candidate for antifibrotic therapy and presents a universily applicable PROTAC design method utilizing both ends of this heterobifunctional molecule, leading to the much better biological activities.

## Methods

### In vitro ubiquitination assay

Fresh whole cell lysates of ACHN cell lines were used to detect the activity of PROTAC on Smad3 degradation via ubiquitin–proteasome pathway in vitro [50]. The cell lysates were quantified with BCA assay (Thermo Fisher Scientific, USA). Smad3 recombinant protein (HY-P71323, MedChemExpress, USA) and PROTAC in different concentrations (0, 1, 5, 10 μg) were added to the cell lysates in a water bath at 37 °C for 30 min. Then 33 μL of 4$\times$ loading buffer was added to 100 μL of this reaction mixture. The sample was boiled for 10 min before western blot analysis. To further explore weather the degradation of Smad3 was through ubiquitin proteasome pathway, 5 μM or 10 μM MG132 (proteasome inhibitor, MedChemExpress, USA) were added into the cell lysates too. Then the protein level of Smad3, VHL and ubiquitin were all detected by western blot.

### Cell culture and treatments

Normal rat kidney fibroblast cells (NRK-49F, ATCC, USA), human renal proximal tubule cells (HK2, ATCC, USA), mouse renal proximal tubule cells (mTEC, ATCC, USA) and rat renal proximal tubule cells (NRK-52E, ATCC, USA) were cultured at 37 °C in 5% CO2 in DMEM mixed 1:1 (vol./vol.) with F12 medium (Life Technologies) and 10% FBS (Gibco). Cells were grown to approximately 70–80% confluence and stimulated with PROTAC of different linkers (PROTAC-PEG, PROTAC-(CH_2_COOH)_2_) and PROTAC with different chiralities (PROTAC-R, PROTAC-S) in serum-free medium. To determine the effect of PROTAC on target proteins in vitro, PROTAC was directly added to NRK-49F and HK2 separately.

### In vivo toxicity and pharmacokinetic evaluation

For subacute and chronic toxicity evaluation, ICR mice (6–8 weeks) were divided into 8 groups (n = 5) in random: male/female control, low-dose (50 mg/kg), middle-dose (150 mg/kg) and high-dose group (300 mg/kg) respectively. All mice were administered subcutaneous injection (s.c.) with solvent/PROTAC for 7 consecutive days. Then 3 mice of each group were dissected for evaluation of subacute toxicity. The remaining 2 mice of each group were under observation for 7 days without treatment for evaluation of chronic toxicity.

To clarify the mode of administration for in vivo testing, PROTAC were administrated i.v. (2 mg/kg), i.p. (5 mg/kg), s.c. (5 mg/kg) or i.g. (10 mg/kg) to ICR mice separately and the blood samples were collected at 0, 5, 15, 30, 60, 120 and 240 min (n = 3/per group), using the Liquid chromatography-tandem mass spectrometry (LC–MS/MS) to determine the plasma concentration. Tissues concentration of PROTAC was also measured at 4, 8, 24 h after s.c. (5 mg/kg) administration (n = 5/per group).

### Animals experimental protocols

Renal interstitial fibrosis was induced by unilateral ureteral obstruction (UUO) surgery as previously described with adult male C57BL/6 J mice (6–8 weeks) [51]. Mice were randomly divided into four groups (n = 6/each group): Sham + Vehicle, Sham + PROTAC, UUO + Vehicle, UUO + PROTAC. Vehicle/PROTAC (Ontores, Zhejiang, China) was given by i.p. twice daily in the dosage of 30 mg/kg for 5 days before surgery and 90 mg/kg for another 9 days after the surgery (Fig. 4A).

Chronic renal failure (CRF) was induced by a two stage 5/6 nephrectomy model (5/6Nx) as described before [52]. Mice were randomly divided into four groups (n = 8/each group): Sham + Vehicle, Sham + PROTAC, 5/6Nx + Vehicle, 5/6Nx + PROTAC. After surgery, Vehicle/PROTAC was given s.c. every 2 days in the dosage of 24 mg/kg for 5 weeks (Fig. 5A). Blood was collected under isoflurane anesthesia every week for hematological and biochemical studies. All Mice were sacrificed 4 h after the final injection. Studies were approved by the Sun Yat-Sen University Institutional Animal Care and Use Committee (No. SYSU-IACUC-2018-000280).

### Immunoblot analysis

Protein was extracted from renal tissues and cells using the radio immunoprecipitation assay (RIPA) lysis buffer with protease inhibitor cocktail (Roche, Switzerland). A total of 50 μg protein was subjected to SDS‐PAGE (8–16%) gel and then transferred onto polyvinylidene fluoride membranes. Primary antibodies used in this study were as follows: Smad3 (ab40854), HIF-2$\alpha$(ab199), HIF-1$\alpha$ (ab1, ab179483), Collagen-I (ab6308), HAF (ab181867), RACK1 (ab129084) and VHL (ab77262) purchased from Abcam (UK), $\alpha$-SMA (A5228) purchased from sigma (USA), CHIP (#2080) purchased from CST (USA) and Ubiquitin (PTM-5789) purchased from PTM BIO (China). Detection was carried out with the ECL system, and signals were revealed and quantified against the internal loading control with ChemiScope 6200 Touch (Clinx Science Instruments, Shanghai, China).

### Real-time quantitative PCR

Total RNA was isolated from the kidney, liver and duodenum tissues using Trizol (Invitrogen, CA) according to the manufacturer’s instructions. Real-time qPCR was performed by SYBR Green Supermix using CFX96 PCR System (Bio-Rad, CA). The indicated primers were listed in Additional file 2: Table S4.

### Renal histology, immunohistochemistry and blood analysis

Both histological and immunohistochemical analyses were performed on the formalin fixed, paraffin-embedded tissue Sects. (5 μm). Collagen deposition in renal tissue was evaluated by Masson’s trichrome staining (MTS, HT15, Sigma, USA) according to the manufacturer’s instructions. Ten consecutive fields of tubulointerstitium were assessed at $\times$400 magnification in blinded-side (Axioplan2 imaging, Carl Zeiss, Oberkoche, Germany) and the quantification of Masson positive staining area was analyzed with Image J program (http://www.imagej.nih.gov/ij/) as previously reported [53]. For immunohistochemistry (IHC), tissue sections were incubated with primary antibodies against F4/80 (ab16911, Abcam, UK), CD34 (ab81289, Abcam, UK) and developed with diaminobenzidine (DAB) to produce a brown color. Ten consecutive fields excluding small vessels and glomeruli were taken in a blinded fashion under $\times$400 magnification and macrophages stained with F4/80 and the peritubular capillaries stained with CD34 were annotated and counted per high field as previously reported [23, 54].

Serum vascular endothelial growth factor (VEGF, MMV00, R&D Systems, USA), erythropoietin (EPO, MEP00B, R&D Systems, USA), ferritin (ab157713, Abcam, UK), hepcidin (CSB-E14395m, CUSABIO, China), Iron (Fe^2+^, KA1647, Abnova, USA) and cystatin C (ab201280, Abcam, UK) levels were determined with a commercially available ELISA kit according to the manufacturer’s instructions. Hematological data were assessed in whole blood with heparin with BC-5000 Vet (Mindray, Shenzhen, China). Serum biochemical data were analyzed through automatic method and equipment (Hitachi 717 Chemistry Analyzer, Tokyo, Japan).

### Statistical analysis

Data were presented as mean ± SD. All analyses were performed with GraphPad Prism version 8.0 (GraphPad Software, San Diego, CA). Student’s *t*-test was used for statistical comparisons between two groups and one-way analysis of variance (ANOVA) followed by Tukey’s post-test were used for three groups or more. *P* values < 0.05 were considered statistically significant.

## Supplementary Information

**Additional file 1. Fig. S1**: Molecular docking of PROTAC with crystal structures of Smad3 and VHL. **A** Hydrogen bond interactions between amino acid residues SER263, THR246, HIS248 of Smad3 (PDB code: 1mk2) and PROTAC. **B** Hydrogen bond interactions between amino acid residues ARG107, HIS110, SER111, TYR98, HIS115 of VHL (PDB code: 1mk2) and PROTAC. **Fig. S2**: Confirmation of the purity and the structure of PROTAC. **A** The purity of PROTAC was confirmed with High Performance Liquid Chromatography (HPLC). **B**, **C** Full-length sequence of synthesized PROTAC was verified by mass spectrometry. **Fig. S3**: Western blot analyses of HIF-1α, HIF-2α and Smad3 expression in UUO animal model. **Fig. S4**: The effects of PROTAC on the transcriptional level of HIF-1α, HIF-2α and Smad3 in UUO and 5/6Nx animal model. The mRNA expression level of HIF-1α, HIF-2α and Smad3 was measured with real-time qPCR in vehicle or PROTAC-treated mice of A UUO model and B 5/6Nx model (n = 5–8/per group, Data are means ± SD. NS = not significant, *P < 0.05, ****P < 0.0001. Veh = vehicle, UUO = unilateral ureteral obstruction, 5/6Nx= 5/6 nephrectomy model). **Fig. S5**: The effects of PROTAC on the transcriptional level of target genes of HIF-α in duodenum, liver and kidney of normal and 5/6Nx mice. The mRNA expression level of **A** duodenal cytochrome b (Dcytb), **B** divalent metal transporter1 (DMT1), **C** Ferroportin1 (FPN1) in duodenum and **D** transferrin, **E** transferrin receptor1 (TfR1) in liver and **F** EPO in kindey was measured with real-time qPCR in vehicle or PROTAC-treated normal and 5/6Nx mice (n = 4–6/per group, Data are means ± SD. NS = not significant, *P < 0.05, **P < 0.01, ***P < 0.001, ****P < 0.0001. Veh = vehicle, 5/6Nx = 5/6 nephrectomy model). **Fig. S6**: The effects of PROTAC on the transcriptional levels of Smad3 target genes. The mRNA expression level of **A** Collagen-I and **B** Fibronectin in vehicle or PROTAC-treated normal and 5/6Nx mice (n = 5–6/per group, Data are means ± SD. **P < 0.01, ***P < 0.001, ****P < 0.0001. Veh = vehicle, 5/6Nx = 5/6 nephrectomy model).

**Additional file 2. Table S1**: Routine blood analysis of mice in PROTAC subacute toxicity assessment. **Table S2**: Biochemical analysis of mice in PROTAC subacute toxicity assessment. **Table S3**: The pharmacokinetics profile of PROTAC in mice. **Table S4**: cDNA primers used in real-time PCR measurements.

## Data Availability

All data needed to evaluate the conclusions in this study are available in the main text and the supplementary materials.
