# Supplementary material for: VHL-recruiting PROTAC attenuates renal fibrosis and preserves renal function via simultaneous degradation of Smad3 and stabilization of HIF-2α
Source: Cell Biosci. 2022 Dec 19;12:203. doi: 10.1186/s13578-022-00936-x (PMC9761961; doi:10.1186/s13578-022-00936-x)
Supplement: Supplementary file 1 — Additional file 1. Fig. S1: Molecular docking of PROTAC with crystal structures of Smad3 and VHL. A Hydrogen bond interactions between amino acid residues SER263, THR246, HIS248 of Smad3 (PDB code: 1mk2) and PROTAC. B Hydrogen bond interactions between amino acid residues ARG107, HIS110, SER111, TYR98, HIS115 of VHL (PDB code: 1mk2) and PROTAC. Fig. S2: Confirmation of the purity and the structure of PROTAC. A The purity of PROTAC was confirmed with High Performance Liquid Chromatography (HPLC). B, C Full-length sequence of synthesized PROTAC was verified by mass spectrometry. Fig. S3: Western blot analyses of HIF-1α, HIF-2α and Smad3 expression in UUO animal model. Fig. S4: The effects of PROTAC on the transcriptional level of HIF-1α, HIF-2α and Smad3 in UUO and 5/6Nx animal model. The mRNA expression level of HIF-1α, HIF-2α and Smad3 was measured with real-time qPCR in vehicle or PROTAC-treated mice of A UUO model and B 5/6Nx model (n = 5–8/per group, Data are means ± SD. NS = not significant, *P < 0.05, ****P < 0.0001. Veh = vehicle, UUO = unilateral ureteral obstruction, 5/6Nx= 5/6 nephrectomy model). Fig. S5: The effects of PROTAC on the transcriptional level of target genes of HIF-α in duodenum, liver and kidney of normal and 5/6Nx mice. The mRNA expression level of A duodenal cytochrome b (Dcytb), B divalent metal transporter1 (DMT1), C Ferroportin1 (FPN1) in duodenum and D transferrin, E transferrin receptor1 (TfR1) in liver and F EPO in kindey was measured with real-time qPCR in vehicle or PROTAC-treated normal and 5/6Nx mice (n = 4–6/per group, Data are means ± SD. NS = not significant, *P < 0.05, **P < 0.01, ***P < 0.001, ****P < 0.0001. Veh = vehicle, 5/6Nx = 5/6 nephrectomy model). Fig. S6: The effects of PROTAC on the transcriptional levels of Smad3 target genes. The mRNA expression level of A Collagen-I and B Fibronectin in vehicle or PROTAC-treated normal and 5/6Nx mice (n = 5–6/per group, Data are means ± SD. **P < 0.01, ***P < 0.001, ****P < 0.0001 [file 13578_2022_936_MOESM1_ESM.pptx]

## Slide 1
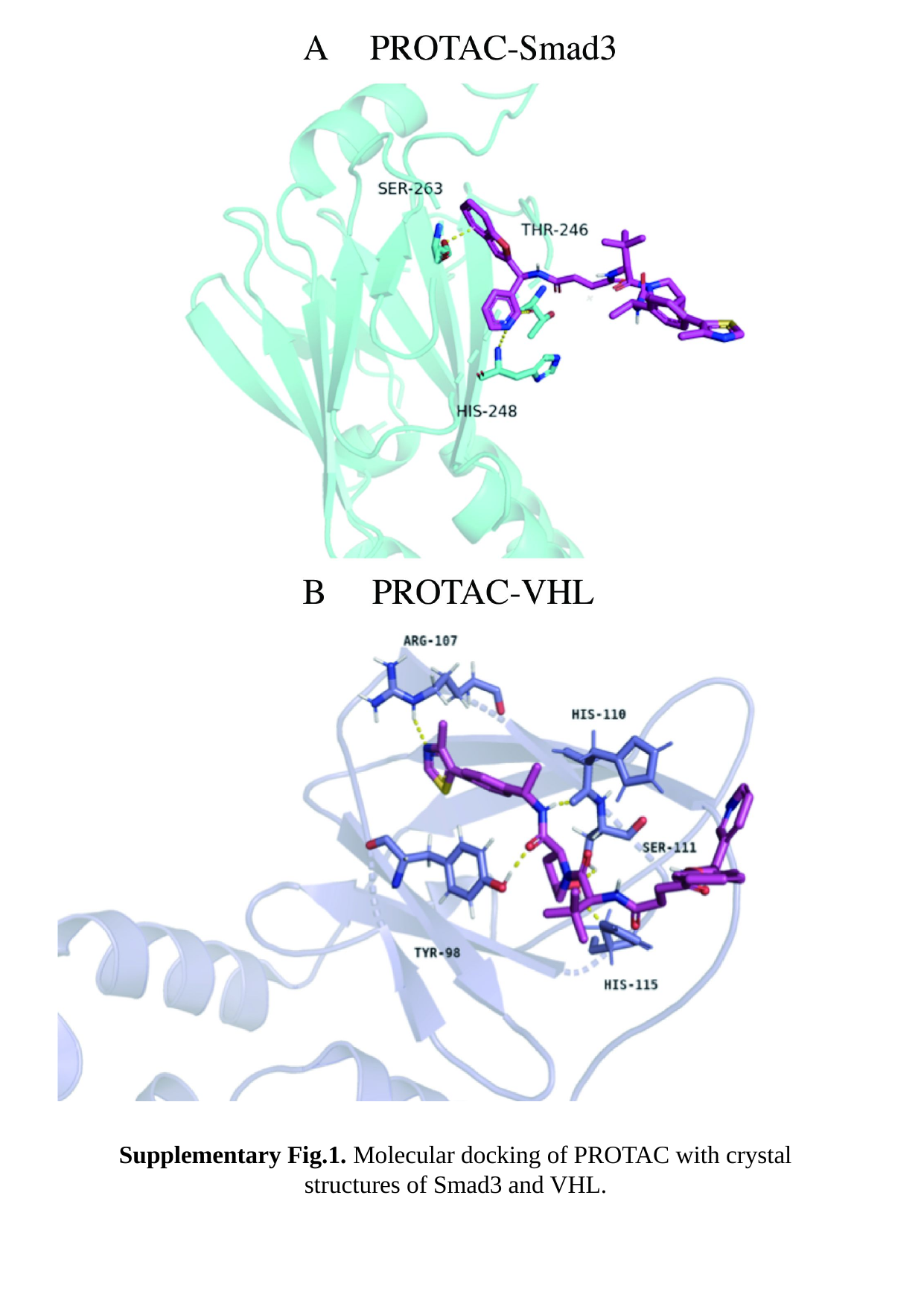

Supplementary Fig.1. Molecular docking of PROTAC with crystal structures of Smad3 and VHL.

## Slide 2
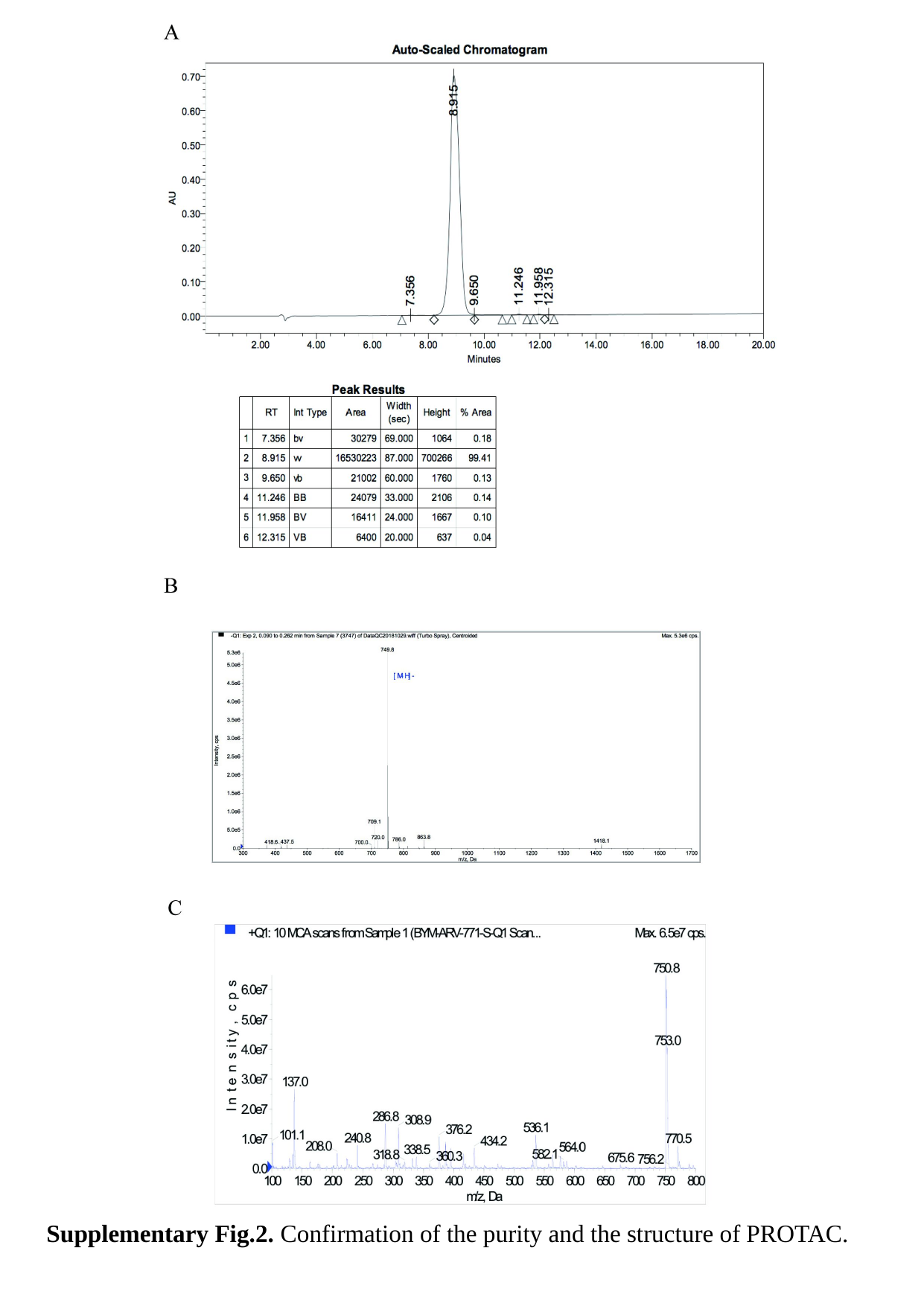

Supplementary Fig.2. Confirmation of the purity and the structure of PROTAC.

## Slide 3
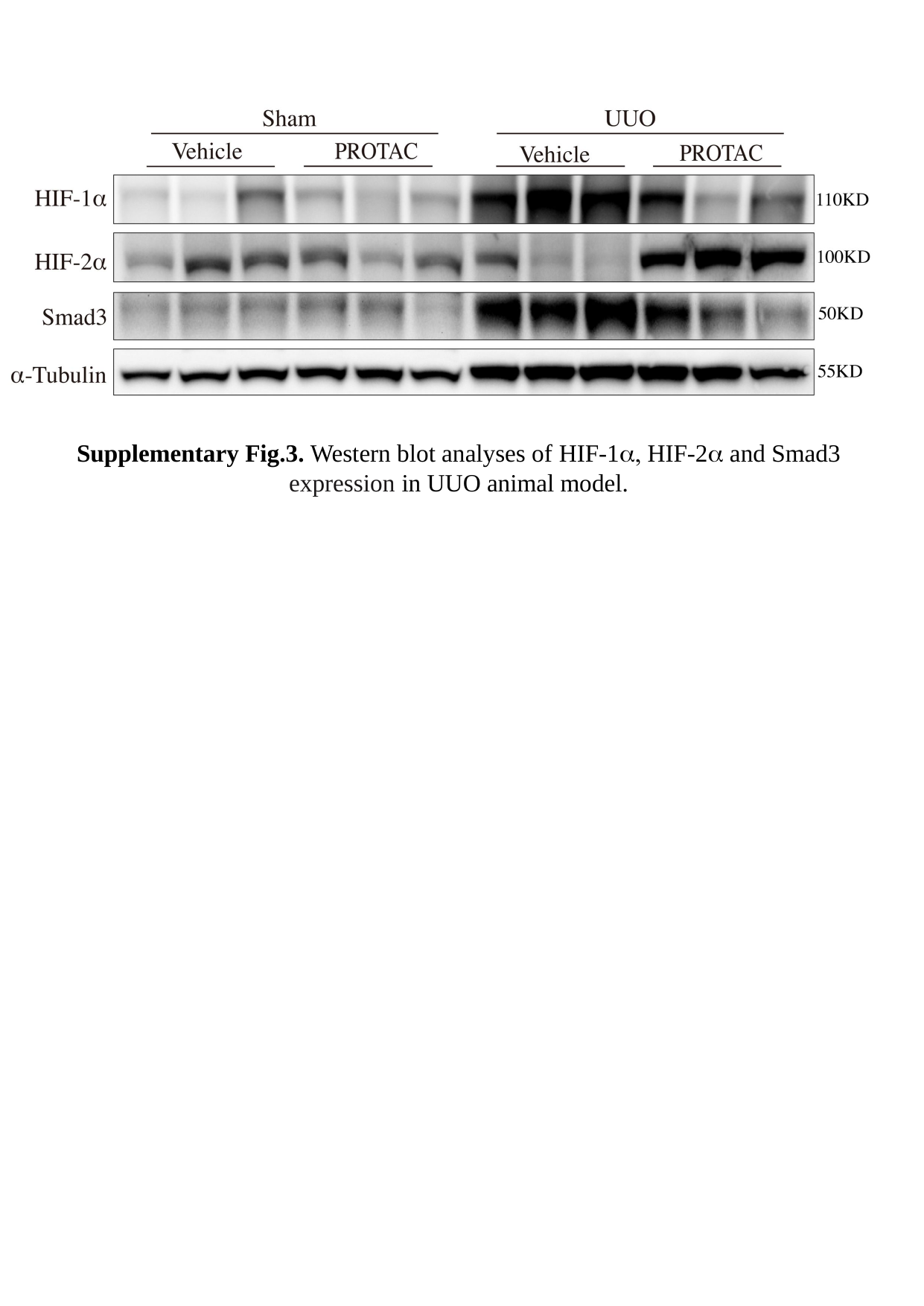

Supplementary Fig.3. Western blot analyses of HIF-1a, HIF-2a and Smad3 expression in UUO animal model.

## Slide 4
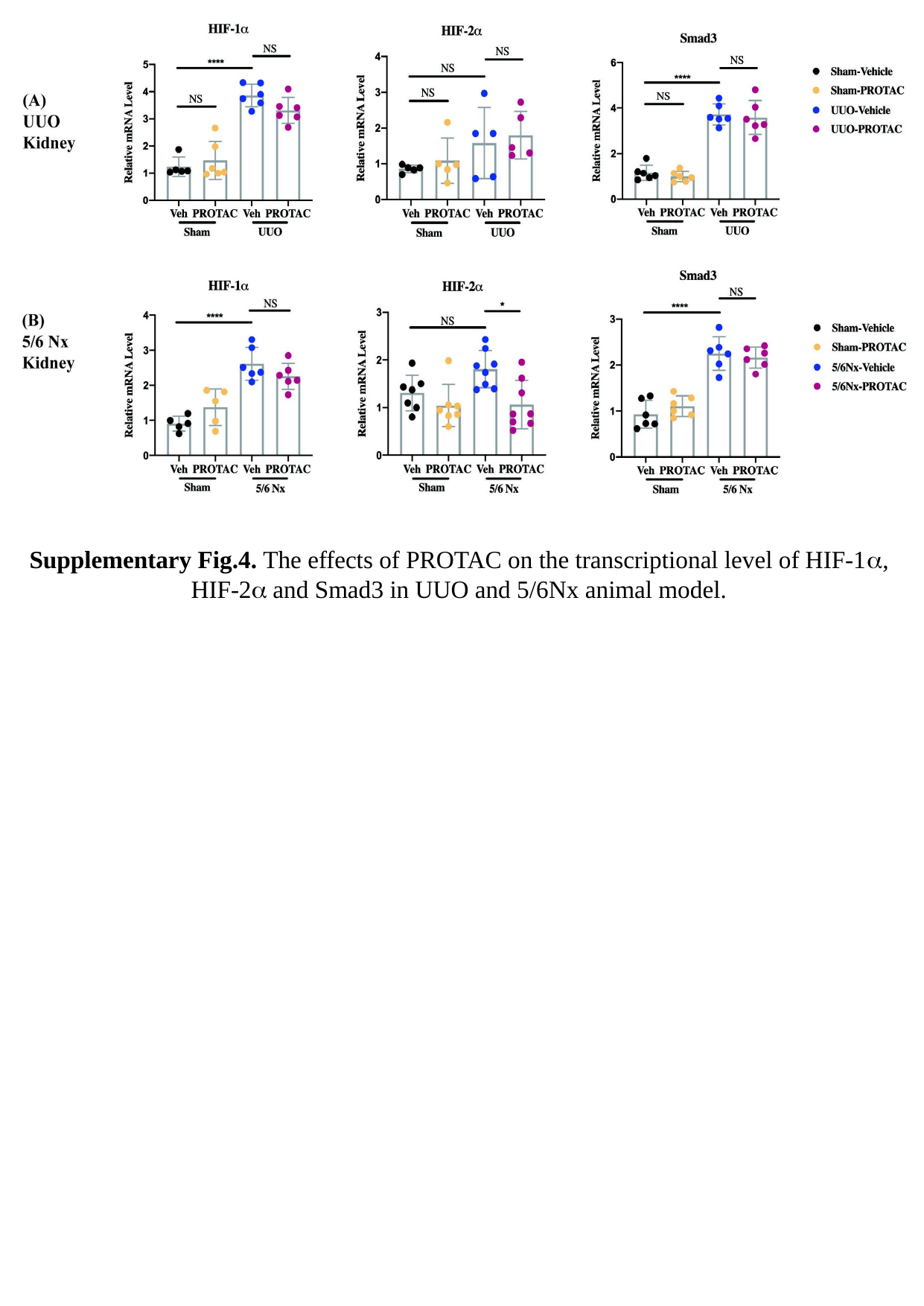

Supplementary Fig.4. The effects of PROTAC on the transcriptional level of HIF-1a, HIF-2a and Smad3 in UUO and 5/6Nx animal model.

## Slide 5
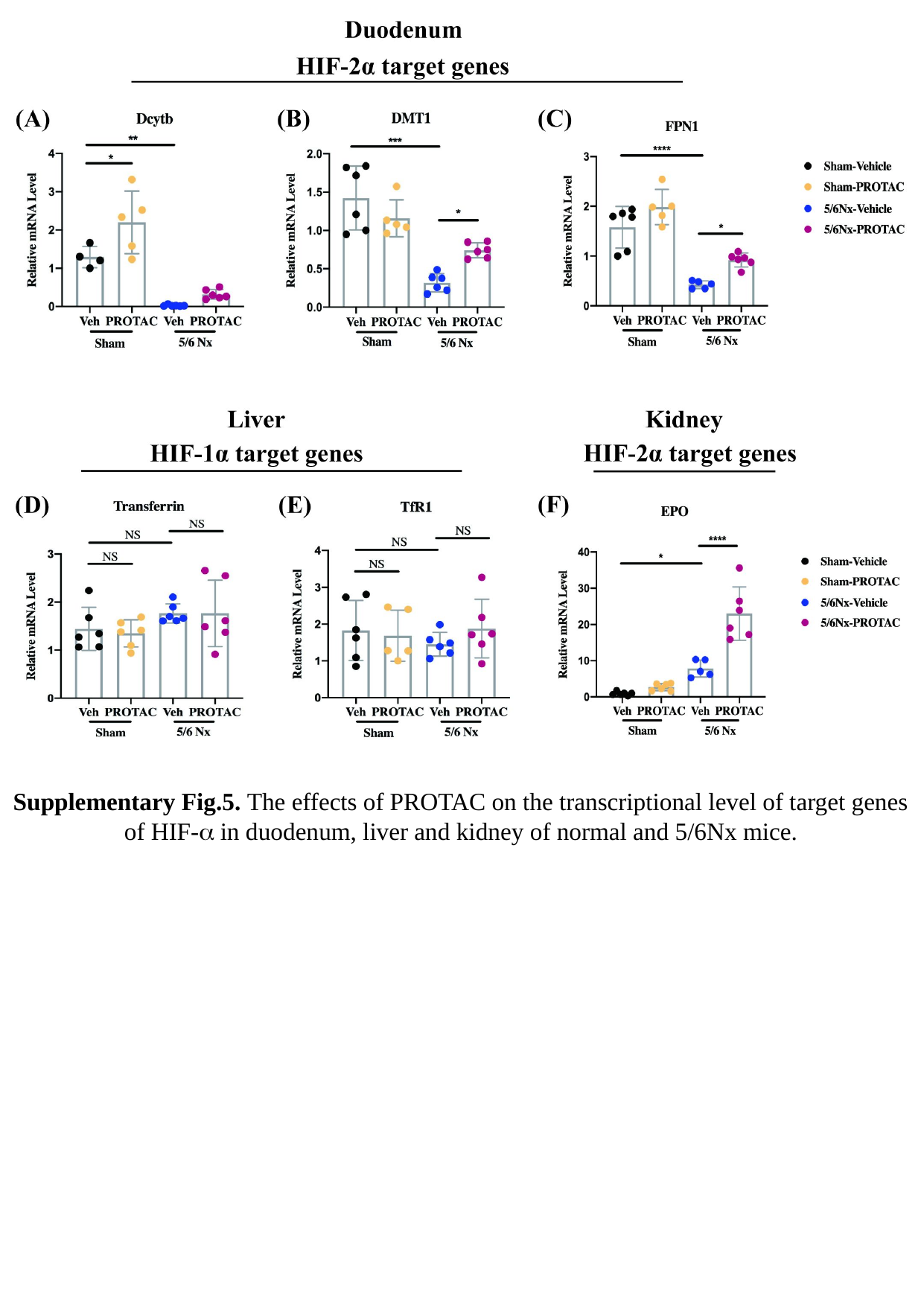

Supplementary Fig.5. The effects of PROTAC on the transcriptional level of target genes of HIF-a in duodenum, liver and kidney of normal and 5/6Nx mice.

## Slide 6
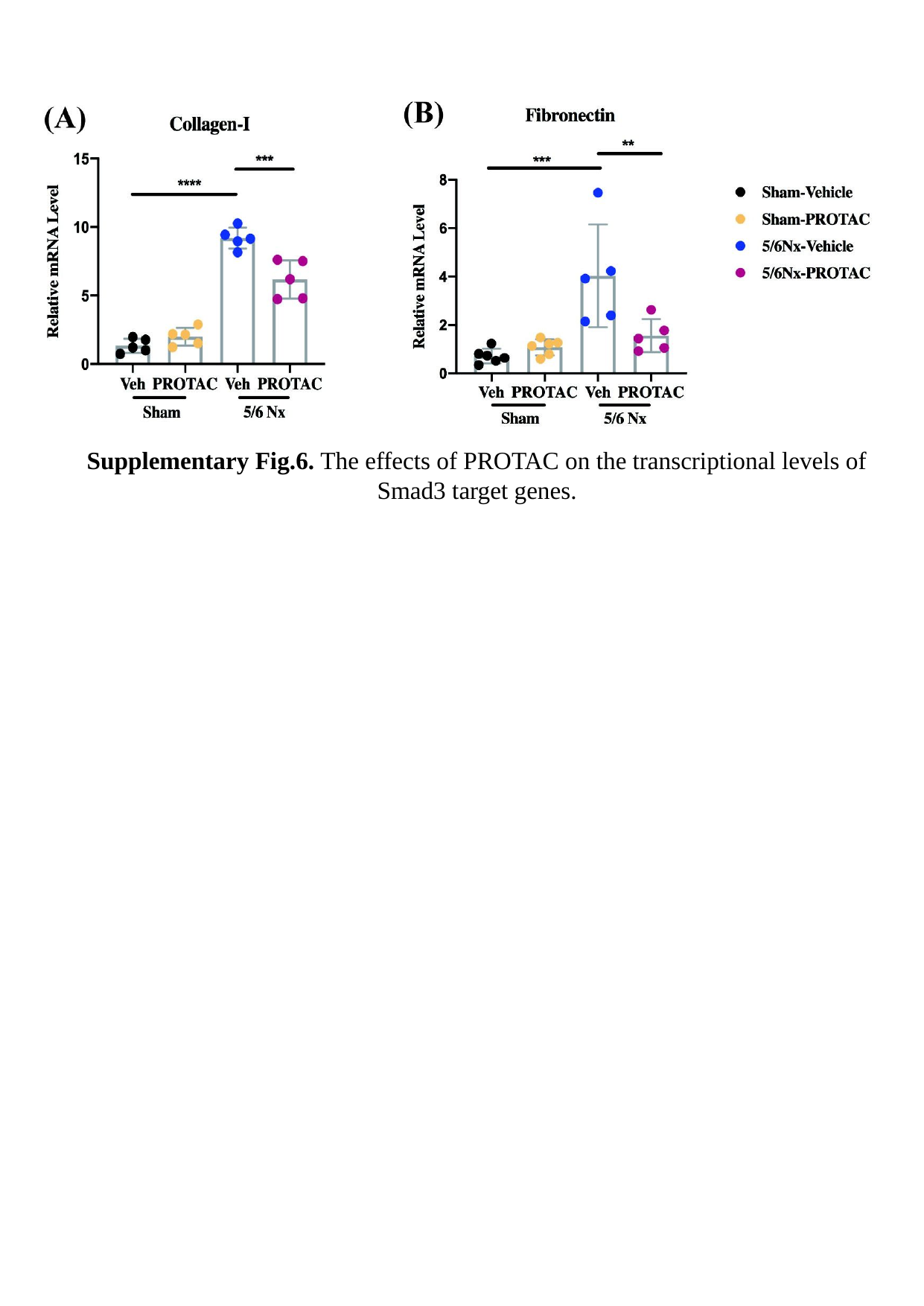

Supplementary Fig.6. The effects of PROTAC on the transcriptional levels of Smad3 target genes.
