# Supplementary material for: VHL-recruiting PROTAC attenuates renal fibrosis and preserves renal function via simultaneous degradation of Smad3 and stabilization of HIF-2α
Source: Cell Biosci. 2022 Dec 19;12:203. doi: 10.1186/s13578-022-00936-x (PMC9761961; doi:10.1186/s13578-022-00936-x)
Supplement: Supplementary file 2 — Additional file 2. Table S1: Routine blood analysis of mice in PROTAC subacute toxicity assessment. Table S2: Biochemical analysis of mice in PROTAC subacute toxicity assessment. Table S3: The pharmacokinetics profile of PROTAC in mice. Table S4: cDNA primers used in real-time PCR measurements. [file 13578_2022_936_MOESM2_ESM.docx]

**Supplementary Table 1.** Routine blood analysis of mice in PROTAC subacute toxicity assessment.

| Clinical Index | Vehicle (n=3) | 50mg/kg (n=3) | 150mg/kg (n=3) | 300mg/kg (n=3) |
| --- | --- | --- | --- | --- |
| **Female** |  |  |  |  |
| HGB (g/L) | 196.0±13.0 | 187.7±6.8 | 180.7±0.6 | 184.0±20.8 |
| RBC (10^12^/L) | 10.1±0.9 | 9.3±0.3 | 9.1±0.4 | 9.0±1.1 |
| HCT (%) | 56.8±10.3 | 50.0±1.9 | 49.2±2.1 | 47.9±3.9 |
| MCV (fL) | 56.0±4.8 | 53.5±1.5 | 54.1±0.2 | 53.4±1.9 |
| MCH (pg) | 19.5±2.3 | 20.1±0.4 | 19.9±0.8 | 20.5±0.1 |
| MCHC (g/L) | 352.0±61.3 | 375.6±4.6 | 367.4±15.1 | 383.5±12.4 |
| PLT (×10^9^/L) | 662.7±173.4 | 733.3±78.0 | 801.7±87.9 | 727.0±158.8 |
| WBC (×10^9^/L) | 5.7±1.9 | 5.3±2.6 | 6.6±0.8 | 6.1±1.7 |
| **Male** |  |  |  |  |
| HGB (g/L) | 185.0±6.0 | 182.7±2.1 | 176.3±10.7 | 183.7±2.5 |
| RBC (10^12^/L) | 9.3±0.4 | 9.1±0.2 | 8.8±0.6 | 9.3±0.3 |
| HCT (%) | 49.5±1.2 | 48.9±0.5 | 47.2±2.6 | 48.8±0.9 |
| MCV (fL) | 53.1±0.9 | 53.6±1.0 | 53.4±0.8 | 52.7±0.9 |
| MCH (pg) | 19.9±0.3 | 20.1±0.7 | 20.0±0.7 | 19.8±0.5 |
| MCHC (g/L) | 374.0±3.2 | 373.6±7.4 | 373.9±8.7 | 376.1±3.0 |
| PLT (×10^9^/L) | 716.0±46.9 | 598.3±106.7 | 746.3±134.2 | 804.0±116.0 |
| WBC (×10^9^/L) | 6.7±0.9 | 9.0±4.0 | 9.3±3.4 | 9.7±4.3 |

Note: Data are presented as mean ± SD. HGB= hemoglobin, RBC= red blood cell count, HCT=hematocrit, MCV=mean corpuscular volume, MCH= mean corpuscular hemoglobin, MCHC= mean corpuscular hemoglobin concentration, PLT=platelet, WBC= white blood cell count.

**Supplementary Table 2.** Biochemical analysis of mice in PROTAC subacute toxicity assessment.

| Clinical Index | Vehicle (n=3) | 50mg/kg (n=3) | 150mg/kg (n=3) | 300mg/kg (n=3) |
| --- | --- | --- | --- | --- |
| **Female** |  |  |  |  |
| ALT (U/L) | 27.3±3.7 | 33.2±1.2 | 31.8±5.9 | 21.9±3.9 |
| AST (U/L) | 116.8±13.1 | 109.6±34.5 | 107.0±16.0 | 107.5±18.8 |
| ALP (U/L) | 221.3±13.2 | 218.8±22.0 | 205.8±11.8 | 201.4±24.6 |
| TP (g/L) | 51.4±1.8 | 49.5±3.3 | 48.5±1.9 | 46.9±2.9 |
| ALB (g/L) | 31.2±0.9 | 29.8±2.1 | 28.9±0.9 | 28.5±0.7 |
| TG (mmol/L) | 1.4±0.3 | 1.5±0.1 | 1.3±0.3 | 1.0±0.2 |
| TC (mmol/L) | 2.5±0.2 | 2.7±0.4 | 2.9±0.1 | 2.3±0.7 |
| CREA (μmol/L) | 25.0±3.4 | 29.2±4.3 | 27.9±6.0 | 20.9±3.8 |
| UREA (mmol/L) | 7.6±1.1 | 7.2±0.3 | 7.5±1.3 | 6.9±1.9 |
| CK (U/L) | 954.7±226.4 | 1145.7±615.9 | 761.9±324.9 | 610.8±122.3 |
| P (mmol/L) | 2.4±0.2 | 3.1±0.3 | 3.2±0.6 | 2.6±0.1 |
| Ca (mmol/L) | 2.4±0.0 | 2.5±0.1 | 2.5±0.2 | 2.3±0.1 |
| Glu (mmol/L) | 5.9±0.6 | 6.1±0.7 | 6.3±0.4 | 5.2±1.3 |
| **Male** |  |  |  |  |
| ALT (U/L) | 36.5±9.1 | 29.8±5.1 | 33.2±10.3 | 34.4±12.4 |
| AST (U/L) | 113.3±32.7 | 106.4±8.0 | 133.8±68.8 | 171.7±33.0 |
| ALP (U/L) | 219.3±12.6 | 195.9±44.6 | 207.0±70.0 | 169.9±25.2 |
| TP (g/L) | 50.9±1.7 | 50.3±5.0 | 49.9±2.8 | 51.3±0.9 |
| ALB (g/L) | 29.5±1.0 | 30.1±3.3 | 26.5±2.1 | 30.2±0.8 |
| TG (mmol/L) | 1.3±0.5 | 1.2±0.1 | 0.9±0.1 | 0.9±0.3 |
| TC (mmol/L) | 3.4±0.2 | 3.3±0.3 | 3.9±0.4 | 3.3±0.1 |
| CREA (μmol/L) | 27.6±3.1 | 25.1±5.9 | 20.4±1.5 | 28.6±4.1 |
| UREA (mmol/L) | 8.3±0.4 | 8.4±0.4 | 7.3±0.8 | 8.1±1.3 |
| CK (U/L) | 1194.6±415.7 | 1177.0±352.2 | 1375.5±758.4 | 1748.0±418.4 |
| P (mmol/L) | 2.9±0.1 | 3.0±0.4 | 3.0±0.3 | 2.9±0.1 |
| Ca (mmol/L) | 2.4±0.1 | 2.4±0.1 | 2.4±0.1 | 2.4±0.1 |
| Glu (mmol/L) | 6.8±1.0 | 5.8±1.3 | 6.6±0.6 | 6.4±0.6 |

Note: Data are presented as mean ± SD. ALT=alanine aminotransferase, AST= aspartate aminotransferase, ALP= alkaline phosphatase, TP=total protein, ALB=albumin, TG=triglyceride, TC= total cholesterol, CREA=creatinine, UREA=urea, CK= creatine kinase, P= phosphorus, Ca=calcium, Glu= glucose.

**Supplementary Table 3.** The pharmacokinetics profile of PROTAC in mice.

| Parameters | i.p. (5mg/kg) | s.c. (5mg/kg) |
| --- | --- | --- |
| T_max_ (h) | 0.25 | 0.5 |
| C_max_ (ng/mL) | 94.1 | 221 |
| AUC_all_ (ng*h/mL) | 90.3 | 613 |
| AUC_inf_ (ng*h/mL) | 90.8 | 633 |
| T_1/2_ (h) | 0.481 | 1.58 |
| MRT_inf_ (h) | 1.04 | 2.34 |

**Note:** T_max_= peak time；C_max_= peak concentration；AUC= area under curve；T_1/2_= half-life；MRT= mean residence time.

**Supplementary Table 4.** cDNA primers used in real-time PCR measurements.

| Genes | | Primer Sequence |
| --- | --- | --- |
| *Smad3* | Forward: 5’- AGG AGA AGT GGT GCG AGA AG -3’ | |
|  | Reverse: 5’- CCA TCC AGT GAC CTG GGG AT -3’ | |
| *HIF-2*$\alpha$ | Forward: 5’- TCC TGT CCT CAG TCT GCT CT -3’ | |
|  | Reverse: 5’- CAT GTC ACC GTC TTG GGT CA -3’ | |
| *HIF-1*$\alpha$ | Forward: 5’- AGG ATG AGT TCT GAA CGT CGA AA -3’ | |
|  | Reverse: 5’- GGG GAA GTG GCA ACT GAT GA -3’ | |
| *DMT1* | Forward: 5’- AAA GAT GCC AGA CGA TGG CG -3’ | |
|  | Reverse: 5’- CTC CAG TGG AAT GTG GGA GG -3’ | |
| *FPN1* | Forward: 5’- ACC AAG GCA AGA GAT CAA ACC -3’ | |
|  | Reverse: 5’- CAA AGT GCC ACA TCC GAT CC -3’ | |
| *Dcytb* | Forward: 5’- ACA GTG ATT GCG ACG GTT CT -3’ | |
|  | Reverse: 5’- GAT GAG GGC TCC AAA CAC CA -3’ | |
| *TfR1* | Forward: 5’- TGG GTC TAA GTC TAC AGT GGC -3’ | |
|  | Reverse: 5’- AGA TAC ATA GGG CGA CAG GAA -3’ | |
| *Transferrin* | Forward: 5’- CGT TAA ACT TCC AGA GGG TAC CAC -3’ | |
|  | Reverse: 5’- CTG TCT CCA CCA CAG TGG CAA CC -3’ | |
| *EPO* | Forward: 5’- CCT GCT AGC CAA TTC CTC CC -3’ | |
|  | Reverse: 5’- CCA GTA CCC GAA GCA GTG AA -3’ | |
| *Collagen-I* | Forward: 5’- TGA CTG GAA GAG CGG AGA GT -3’ | |
|  | Reverse: 5’- GTT CGG GCT GAT GTA CCA GT -3’ | |
| *Fibronectin* | Forward: 5’- ATG TGG ACC CCT CCT GAT AGT -3’ | |
|  | Reverse: 5’- GCC CAG TGA TTT CAG CAA AGG -3’ | |
